# Comparison of autograft and allograft tendons in posterior cruciate ligament reconstruction: A meta-analysis

**DOI:** 10.1097/MD.0000000000007434

**Published:** 2017-07-07

**Authors:** Peng Tian, Wen-qing Hu, Zhi-jun Li, Xiao-lei Sun, Xin-long Ma

**Affiliations:** a Department of Orthopedics, Tianjin Hospital; b Department of Rehabilitation; c Department of Orthopedics, General Hospital of Tianjin Medical University; d Department of Orthopedics Institute, Tianjin Hospital, Tianjin, People's Republic of China.

**Keywords:** allograft, autograft, meta-analysis, posterior cruciate ligament, reconstruction

## Abstract

**Background:**

The purpose of this meta-analysis of randomized controlled trials (RCTs) and non-RCTs was to compare the clinical outcomes of autograft versus allograft tendons in patients who underwent posterior cruciate ligament (PCL) reconstruction.

**Methods:**

We conducted a search of PubMed, EMBASE, The Cochrane Library, and Web of Science databases for RCTs and non-RCTs comparing autograft and allograft tendons in PCL reconstruction up to August 2016. The outcomes were Lysholm knee function score, postoperative objective and subjective International Knee Documentation Committee Score (IKDCS), Tegner activity scale, and knee posterior stability. Data analysis was performed using RevMan 5.3 software.

**Results:**

One RCT and 4 non-RCTs met the inclusion criteria. The current meta-analysis indicated that there were no significant differences in the Lysholm knee function score (mean difference [MD] = −0.99, 95% confidence interval [CI]: −5.51 to 3.54, *P* = .67), Tegner activity scale (MD = 0.46, 95% CI: 0.03 to 0.90, *P* = .04), postoperative objective IKDCS (odds ratio [OR] = 1.66, 95% CI: 0.77 to 3.58, *P* = .20), postoperative subjective IKDCS (MD = 3.00, 95% CI: −0.29 to 6.29, *P* = .07), or knee posterior stability (MD = −0.45, 95% CI: −1.28 to 0.38, *P* = .29) between patients who received autograft tendons and those who received allograft tendons. The patients with autograft tendons had a higher Tegner activity scale (MD = 0.46, 95% CI: 0.03 to 0.90, *P* = .04) than those with allograft tendons.

**Conclusions:**

The present meta-analysis shows that there was insufficient evidence to indicate that allograft tendons were significantly better than autograft tendons for PCL reconstruction. Due to the limited quality and data in the studies currently available, in the future, more high-quality RCTs are required to answer this question more definitively.

## 1 Introduction

With the development of society, sports injuries and traffic accident injuries are gradually increasing, and posterior cruciate ligament (PCL) injuries have become common.^[1,2]^ The PCL, which contains the anterolateral (AL) and posteromedial (PM) bundles, plays an important role in maintaining the stability of the knee joint. PCL rupture can lead to meniscal and articular cartilage damage, which can accelerate degeneration of the knee joint.^[2–4]^ PCL reconstruction is the primary means of treatment for PCL rupture and can delay the progression of knee osteoarthritis.^[5]^

Currently, it has been reported that outcomes of PCL reconstruction vary and need to be improved. One of the important controversies in PCL reconstruction is graft tissue selection between autograft tendons and allograft tendons.^[6–8]^ Autogeneic and allogeneic graft tissues have unique advantages and disadvantages.^[9]^ Li et al^[10]^ demonstrated that the outcomes were similar between hamstring tendon autografts and tibialis anterior allografts in single-bundle PCL reconstruction. Ahn et al^[11]^ reported that the double-loop hamstring tendon autograft was as good as the Achilles tendon allograft for PCL reconstruction. Hashemi-Motlagh et al^[12]^ confirmed that the patients who received autograft or allograft tendons had satisfactory outcomes after surgery but that the postoperative posterior stability of the knee in patients with autologous tendon transplantation was better than those with allogeneic tendon transplantation.

Although both forms of tendons are often used in PCL reconstruction, controversies over their efficacy and safety still exist. The purpose of the present meta-analysis is to compare the effects of autograft versus allograft tendons for PCL single-bundle reconstruction in patients from comparative studies and to provide a reference for the clinical treatment of PCL reconstruction.

## 2 Methods

### 2.1 Inclusion and exclusion criteria

Studies were included if the following criteria were met: study design: comparative studies (randomized controlled trial [RCTs] or non-RCTs); study object: adult patients with PCL tears; operative intervention: patients in the allograft group received allograft tendons for arthroscopic PCL single-bundle reconstruction, and patients in the autograft group received autograft tendons for arthroscopic PCL single-bundle reconstruction; and outcome measures: knee posterior stability, subjective outcome (Lysholm knee function score or Tegner activity scale), the International Knee Documentation Committee Scores (IKDCS, both objective and subjective), and complications.

Exclusion criteria were as follows: open or repeat PCL reconstructive surgery; other ligamentous injuries of the contralateral knee; the literature contained no associated data; or review articles.

### 2.2 Search strategy

According to the Cochrane Collaboration guidelines, we searched Medline (1966–2016.8), PubMed (1966–2016.8), Cochrane library (1966–2016.8), EMBASE (1966–2016.8), and Science Direct (1985–2016.8) for comparative studies comparing autograft with allograft tendons for PCL single-bundle reconstruction. The search terms were as follows: posterior cruciate ligament, allograft, autograft, and reconstruction. No restrictions were imposed on language, regions, or publication type. In addition, the reference lists of all included studies were manually searched to identify trials that may have been missed. Each included study was published in a peer-reviewed journal as a full article, excluding the gray literature and conference proceedings. The search of titles and abstracts was conducted independently by 2 reviewers. Disagreements were resolved by consulting a third reviewer. This study is a meta-analysis, which did not require the ethics committee or institutional review board to approve the study.

### 2.3 Quality assessment

Two reviewers independently evaluated the bias risk of the included studies. RCTs were assessed with the RCT bias risk assessment tools in the Cochrane Handbook Version 5.2. The items used for the assessment of each study were as follows: adequacy of random sequence generation, allocation concealment, blinding of participants, personnel and outcome assessment, handling of dropouts (incomplete outcome data), selective outcome reporting, and other potential sources of bias. Non-RCTs were assessed with the methodological index for nonrandomized studies (MINORS). The methodological quality score is from 0 to 24. Disagreements were resolved by consensus or consultation with a senior reviewer.

### 2.4 Data extraction

For each eligible study, both reviewers extracted all the relevant data independently. The following information was extracted from each study: study ID (author and publication year); sample size and distributions of age and sex; and outcomes and complications. Any disagreement was resolved by discussion; when no consensus could be achieved, a third reviewer acted as the adjudicator and made the final decision. The original authors were contacted for supplementary information when necessary.

### 2.5 Data analysis and statistical methods

The meta-analysis was conducted with Review Manager software 5.2 for Windows (RevMan Version 5.2; The Nordic Cochrane Center, The Cochrane Collaboration, Copenhagen, Denmark). For continuous outcomes, the mean difference (MD) or standardized mean difference (SMD) and 95% confidence intervals (CIs) are presented. Odds ratio (OR) and 95% CIs were calculated for dichotomous data. A *P* value less than 0.05 was considered statistically significant. Statistical heterogeneity was assessed using a standard χ^2^ test with significance set at a *P* value of .1, which was measured by the *I*^2^ statistic. When *I*^2^>50%, *P* <.1 was considered to be significant heterogeneity. In that case, a random-effects model was applied for data analysis. A fixed-effects model was used when no significant heterogeneity was found. In cases of significant heterogeneity, subgroup analysis was performed to investigate the sources. The source of heterogeneity was investigated by subgroup analysis and sensitivity analysis. A random-effects model was used for heterogeneous data; otherwise, a fixed effect model was used.

## 3 Results

### 3.1 Search results

**Figure 1:**
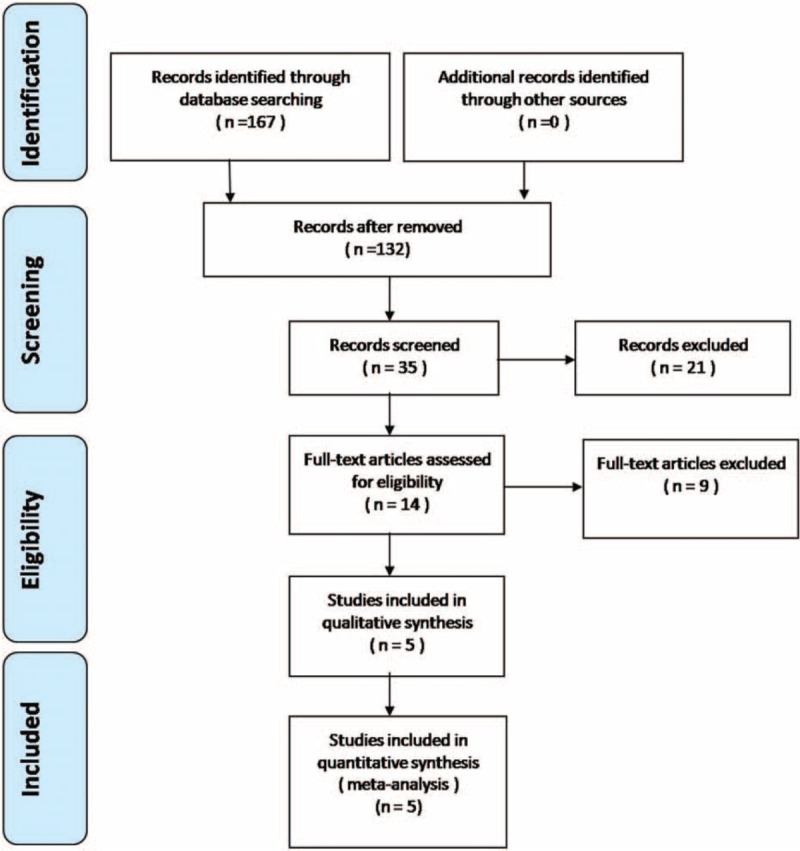
Flowchart of the study selection process.

A total of 167 studies were identified as potentially relevant literature reports. By scanning the titles and abstracts, 153 reports were excluded because of duplication and irrelevancy or because they were case reports, reviews, or not comparative studies. The remaining 14 studies underwent a comprehensive full-text evaluation. No additional studies were obtained after the reference review. Ultimately, 1 RCT and 4 non-RCTs were eligible for data extraction and meta-analysis. The search process is shown in Figure 1.

### 3.2 Study characteristics

The characteristics of the 5 included studies are shown in Table 1. Statistically similar baseline characteristics were observed between both groups such as mean age and gender. All studies had small sample sizes; the number of patients included in the study ranged from 36 to 71 patients. The follow-up period ranged from 2 to 5 years.

**Table 1:**
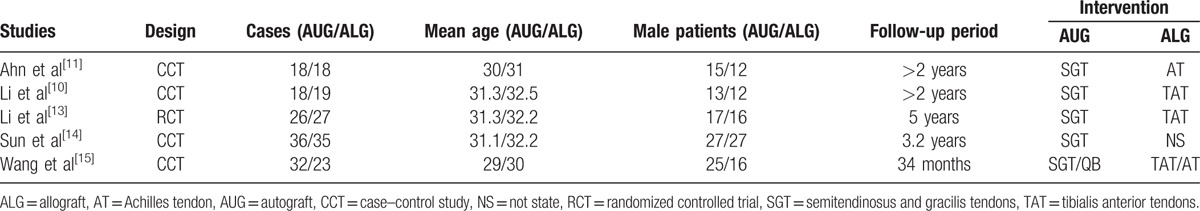
Cohort characteristics.

### 3.3 Risk of bias assessment

**Figure 2:**
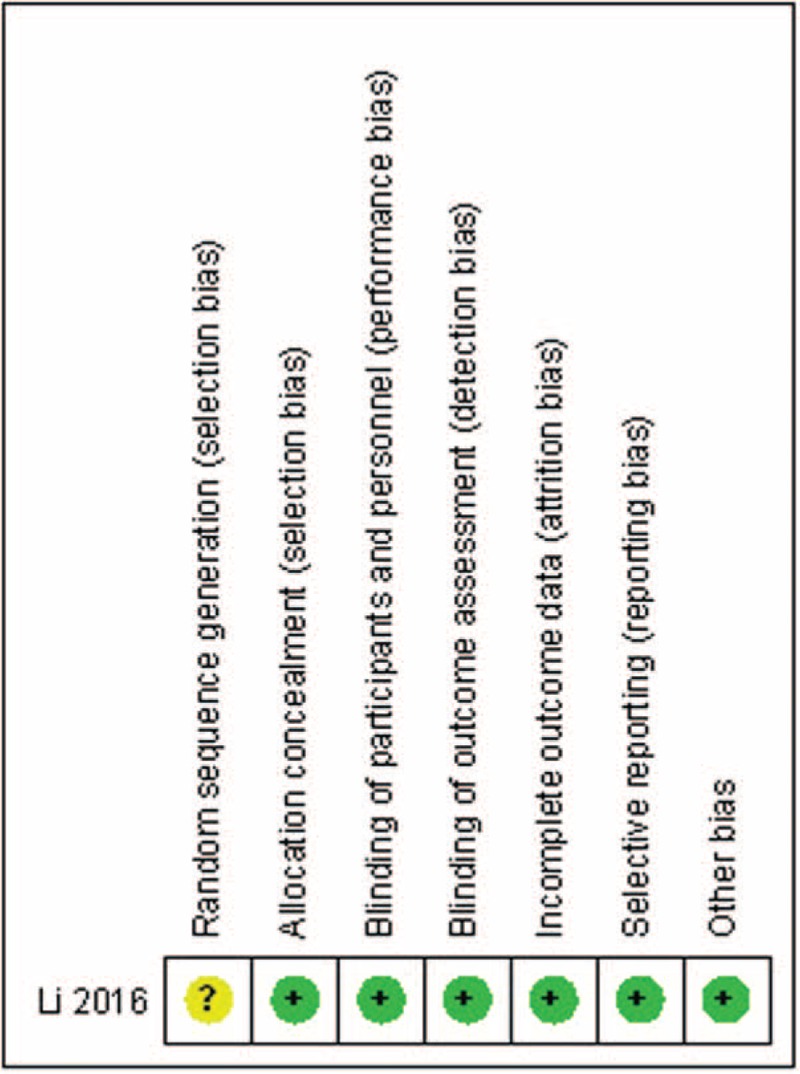
Risk of bias summary.

The RCT quality was assessed based on the Cochrane Handbook for Systematic Review of Interventions (Fig. 2). The RCT stated clear inclusion criteria and did not provide a methodology of randomization; sealed envelopes were used. Blinding of the assessor and participants was provided in the RCT. No unclear bias due to incomplete outcome data or selective outcomes was reported. For the non-RCTs, the MINORS scores were 14 to 17 for the retrospectively controlled trials. The methodological quality assessment is illustrated in Table 2.

**Table 2:**
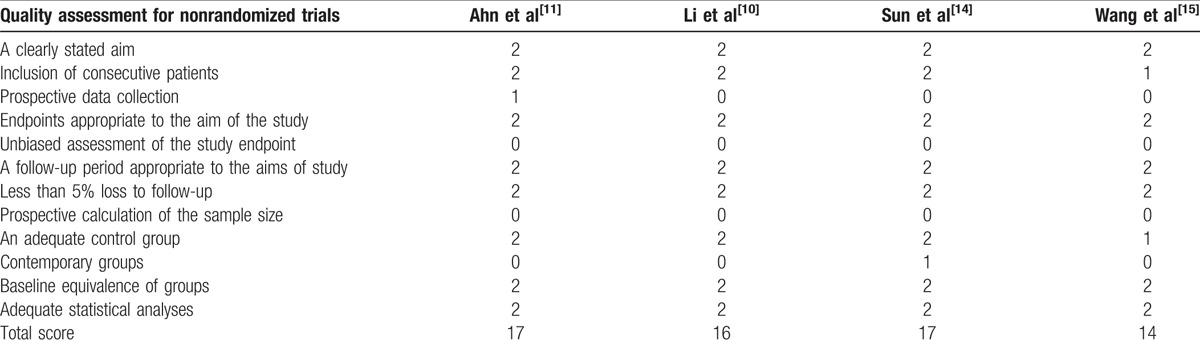
Quality assessment for nonrandomized trials.

### 3.4 Outcomes of the meta-analysis

#### 3.4.1 Lysholm knee function score

**Figure 3:**
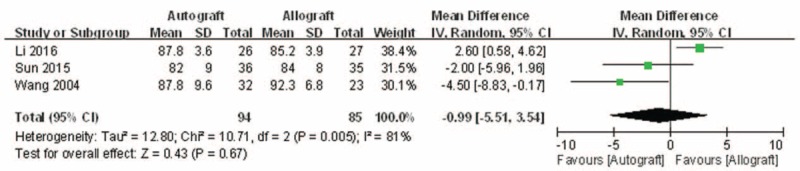
Forest plot showing the Lysholm knee function score.

Lysholm knee function scores were reported in 3 of the studies.^[13–15]^ Significant heterogeneity was found, and a random model was used (*I*^2^ = 81%, *P* = .005). The Lysholm knee function score in the autograft group was not significantly higher than that of the allograft group (MD = −0.99, 95% CI: −5.51 to 3.54, *P* = .67; Fig. 3).

#### 3.4.2 Tegner activity scale

**Figure 4:**
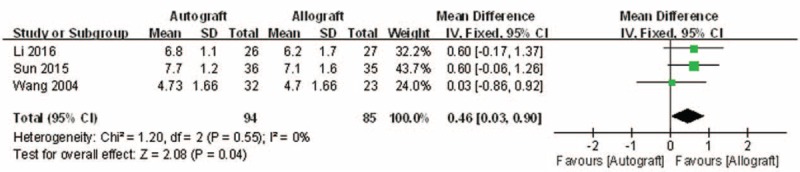
Forest plot showing the Tegner activity scale.

Tegner activity scales were reported in 3 of the studies.^[13–15]^ No significant heterogeneity was found and a fixed-model was applied (*I*^2^ = 0%, *P* = .55). The Tegner activity scale in the autograft group was significantly higher than that of the allograft group (MD = 0.46, 95% CI: 0.03 to 0.90, *P* = .04; Fig. 4).

#### 3.4.3 IKDCS

**Figure 5:**
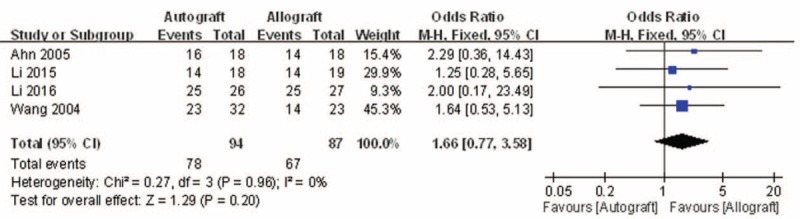
Forest plot showing the postoperative objective IKDCS. IKDCS = International Knee Documentation Committee Score.

Postoperative objective IKDCSs were reported in 4 of the studies.^[10,11,13,15]^ No significant heterogeneity was found, and a fixed-model was applied (*I*^2^ = 0%, *P* = .96). Postoperative objective IKDCSs in the autograft group were not significantly higher than that of the allograft group (OR = 1.66, 95% CI: 0.77 to 3.58, *P* = .20; Fig. 5).

**Figure 6:**
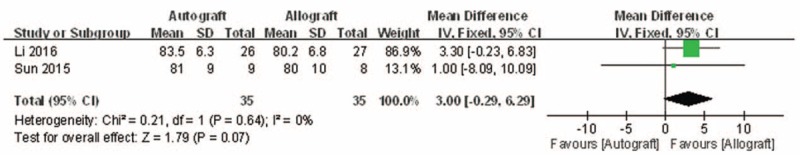
Forest plot showing the postoperative subjective IKDCS. IKDCS = International Knee Documentation Committee Score.

Postoperative subjective IKDCSs were reported in 2 of the studies.^[13,14]^ No significant heterogeneity was found, and a fixed-model was applied (*I*^2^ = 0%, *P* = .64). Postoperative subjective IKDCSs in the autograft group were not significantly higher than that of the allograft group (MD = 3.00, 95% CI: −0.29 to 6.29, *P* = .07; Fig. 6).

#### 3.4.4 Knee posterior stability

**Figure 7:**
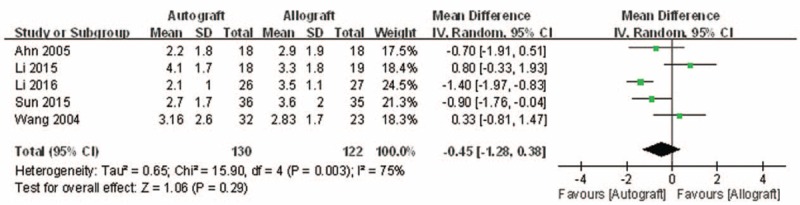
Forest plot showing knee posterior stability.

All 5 studies reported the outcomes of postoperative knee posterior stability, which was defined as posterior translation side-to-side difference.^[10,11,13–15]^ There was significant heterogeneity (*I*^2^ = 75%, *P* = .003); therefore, a random model was performed. Pooling the results demonstrated that postoperative knee posterior stability in the autograft group was not significantly lower than that of the allograft group (MD = −0.45, 95% CI: −1.28 to 0.38, *P* = .29; Fig. 7).

## 4 Discussion

Patients’ postoperative functional recovery and satisfaction determine the overall efficacy of PCL reconstruction. Effective PCL reconstruction can restore knee stability and even delay the progress of osteoarthritis so that patients can achieve better results after rehabilitation.^[16]^ Therefore, it is imperative to choose the appropriate graft for PCL reconstruction. This article focuses on the clinical efficacy of PCL reconstructions using autograft versus allograft tendons. One RCT and 4 non-RCTs were reviewed in the current meta-analysis. The most important finding of this meta-analysis is that the application of an autograft does not bring about more successful outcomes than an allograft.

The present meta-analysis suggests that there were equivalent effects on the Lysholm knee function score in patients who underwent autograft versus allograft PCL reconstruction. The extreme of the 95% CI and the MD of the Lysholm score are quite unlikely to represent an insignificant difference between autograft and allograft PCL reconstruction (MD = −0.99, 95% CI: −5.51 to 3.54). There were similar effects on objective IKDC and subjective IKDC scores in patients who underwent autograft versus allograft PCL reconstruction (OR = 1.66, 95% CI: 0.77 to 3.58 and MD = 3.00, 95% CI: −0.29 to 6.29, respectively). There was no significant difference in knee posterior stability in patients who underwent autograft versus allograft PCL reconstruction (MD = 3.00, 95% CI: −0.29 to 6.29). There was also no difference in functional score between autograft and allograft PCL reconstruction. However, there was a significant difference in the Tegner activity scale in patients who underwent autograft versus allograft PCL reconstruction (MD = 0.46, 95% CI: 0.03 to 0.90). However, the demonstrated difference in the Tegner activity scale associated with the 2 different PCL reconstructions was so small that it is unlikely clinically relevant. On the whole, patients’ postoperative functional recovery and satisfaction were similar between autograft and allograft PCL reconstruction. However, Hashemi-Motlagh et al^[12]^ demonstrated that in the instrumented posterior laxity test, the autograft gave better results than the allograft, but there were no differences in functional scores. These results should be considered when analyzing the present findings.

The best graft source for PCL reconstruction remains controversial.^[13]^ Currently, the graft materials available for PCL reconstruction include autologous tendons, allograft tendons, and artificial ligaments, but the clinical use of artificial ligaments is uncommon.^[7]^ Allograft tendons are widely used in clinical practice,^[10,11,13–15]^ such as Achilles tendons, patellar tendons, hamstring tendons, and anterior tibial tendons. Operations using allograft tendons were shown to have shorter operative times and reduced soft tissue damage, as well as the ability to have grafts of adequate length and diameter. The disadvantages of using allografts are the limited sources of material, high cost, risk of transmissible diseases, and graft rejection. The advantages of autologous tendons include an abundant source of material, no graft rejection, and faster healing. The tendon diameter for PCL reconstruction generally needs to be more than 8 mm, but autologous tendons are relatively smaller, which might lead to limited clinical application in this situation.^[13]^

During the process of clinical application, antigenicity, rejection, and inflammatory responses were encountered with the use of allograft tendons. Sun et al^[14]^ used autogenous hamstring tendons or allograft tendons for PCL reconstruction in 71 patients. The patients in the allograft group had a longer postoperative fever time and significantly higher white blood cell (WBC) counts and neutrophil levels compared with those in the autograft group. The authors reported that both groups of patients had satisfactory outcomes after PCL reconstruction. The autograft gave better results than the allograft in the posterior stability of the knee, but there were no differences in the functional scores between the 2 groups.^[14]^ Of the 5 articles included in the present meta-analysis, only 1 study reported the postoperative fever time following PCL reconstruction, so no meta-analysis of postoperative fever time was performed.^[14]^

In addition, allograft tendons might be likely to transmit disease. The present meta-analysis included 5 studies in which there were no reports of disease transmission in patients receiving allograft tendon transplantation.^[10,11,13–15]^ There were no differences in the functional scores between the gamma-irradiated allograft group and autograft group.^[13,14]^ Thus, allogeneic tendon transplantation can be used to address the insufficiency of autogenous tendons due to multiple ligament injuries and cruciate ligament revision reconstruction.

This study has several potential limitations. First, due to the highly specific nature of the clinical operation, the sample size in each study was relatively small. Second, there were some methodological weaknesses in all of the included studies. Third, as there were many evaluation indexes such as Lysholm score, Tegner score, IKDCS, knee posterior stability, and radiographic examination, some data description methods were inconsistent, and it was not easy to analyze the combined data. Because of the above-mentioned defects and deficiencies, the pooled estimates should be interpreted with caution. Nevertheless, this meta-analysis was conducted by appropriate search strategies, strict inclusion and exclusion criteria, and statistical methods.

## 5 Conclusions

In summary, the present meta-analysis shows that the clinical outcomes were similar between arthroscopic allograft and autograft tendons for PCL reconstruction. Due to the limited quality and data in the studies currently available, in the future, more high-quality RCTs are required to answer this question more definitively.
